# A case of fatal disseminated adenovirus and drug‐resistant Pneumocystis pneumonia in a patient who received chemotherapy for mantle cell lymphoma

**DOI:** 10.1002/ccr3.7220

**Published:** 2023-04-22

**Authors:** Bo Yu, Lakshmi Saravanan, Dena H. Tran, Alexander J. Davies, Harpreet Kaur, Shivakumar Naraynanan, Avelino C. Verceles, Hyeong J. Kim

**Affiliations:** ^1^ Department of Medicine University of Maryland Medical Center Midtown Campus Baltimore Maryland USA; ^2^ American University of Antigua College of Medicine Osbourn Antigua and Barbuda; ^3^ Division of Pulmonary and Critical Care Medicine University of Maryland Medical School of Medicine Maryland Baltimore USA; ^4^ Department of Infectious Diseases University of Maryland Medical Center Midtown Campus Baltimore Maryland USA

**Keywords:** adenovirus infection, Bendamustine, case report, chemotherapy, Cidifovir

## Abstract

Adenovirus (ADV) may cause severe complications in hematopoietic stem cell transplant recipients, but disseminated ADV infections in patients who received chemotherapy alone for hematological malignancies are poorly understood due to the rarity of cases. Concomitant infection with Pneumocystis (PCP) is extremely rare. Despite being diagnostically challenging, a more specific workup needs to be initiated with a low threshold in patients who are exposed to agents with the potential to suppress T cells. We report a fatal case of disseminated ADV and drug‐resistant PCP pneumonia in a patient with mantle cell lymphoma who had only received combination chemotherapy. A 75‐year‐old man who was diagnosed with mantle cell lymphoma 10 months prior was admitted for mild hypoxic respiratory failure. Bendamustine, Rituximab, Cytarabine regimen had resulted in complete remission of his lymphoma, with the last cycle of chemotherapy administered 3 months prior to admission. CT of the chest revealed ground‐glass opacities concerning pneumonia. Initial laboratory tests were remarkable for mild leukopenia. The respiratory viral panel was only positive for ADV. He did not respond to empiric antibiotics for community‐acquired pneumonia and Trimethoprim / Sulfamethoxazole given later for positive Beta D Glucan (BDG) suggestive of Pneumocystis pneumonia. Then, he developed hemorrhagic cystitis, followed by liver and renal function derangement that prompted checking serum ADV viral load by polymerase chain reaction (PCR). This test took 1 week to return, with a viral load of 50, 000 copies/mL suggesting disseminated ADV infection. Despite initiation of Cidofovir, multi‐organ failure continued to progress, and the follow‐up viral load had doubled on Day 2. The patient passed away the same day shortly after transition to comfort care. T cell suppression seems to be a risk factor for disseminated ADV disease. Clinicians may need to maintain a low threshold to send serum quantitative ADV PCR when symptoms are not improved by antimicrobial treatment for more conventional infections in patients who received agents that are known to suppress T cells, such as Bendamustine.

## 1 BACKGROUND

Human adenovirus infection can cause a wide spectrum of diseases in the immunocompetent host that is generally mild and self‐limiting. However, in immunocompromised hosts, adenovirus infection can be severe with progression to multisystem organ failure resulting in mortality as high as 55%.^1^ Disseminated adenovirus infection (DAI) can involve the respiratory tract, gastrointestinal genitourinary systems, myocardium, central nervous system, and eyes.^2^ There is a wide estimate for the incidence of adenovirus infection following hematopoietic stem cell transplants, ranging from 5 to 20%.^3^, ^4^ The immune response to adenovirus is T‐cell‐mediated and allogeneic transplant recipients are at the highest risk of severe disease; however, disseminated disease in adult patients with underlying hematologic malignancy who have not received stem cell transplant seems rare. We report a fatal case of disseminated adenovirus infection and drug‐resistant Pneumocystis pneumonia in an immunocompromised patient with mantle cell lymphoma who had received combination chemotherapy and Rituximab but had not undergone stem‐cell transplantation.

## 2 CASE PRESENTATION

**FIGURE 1:**
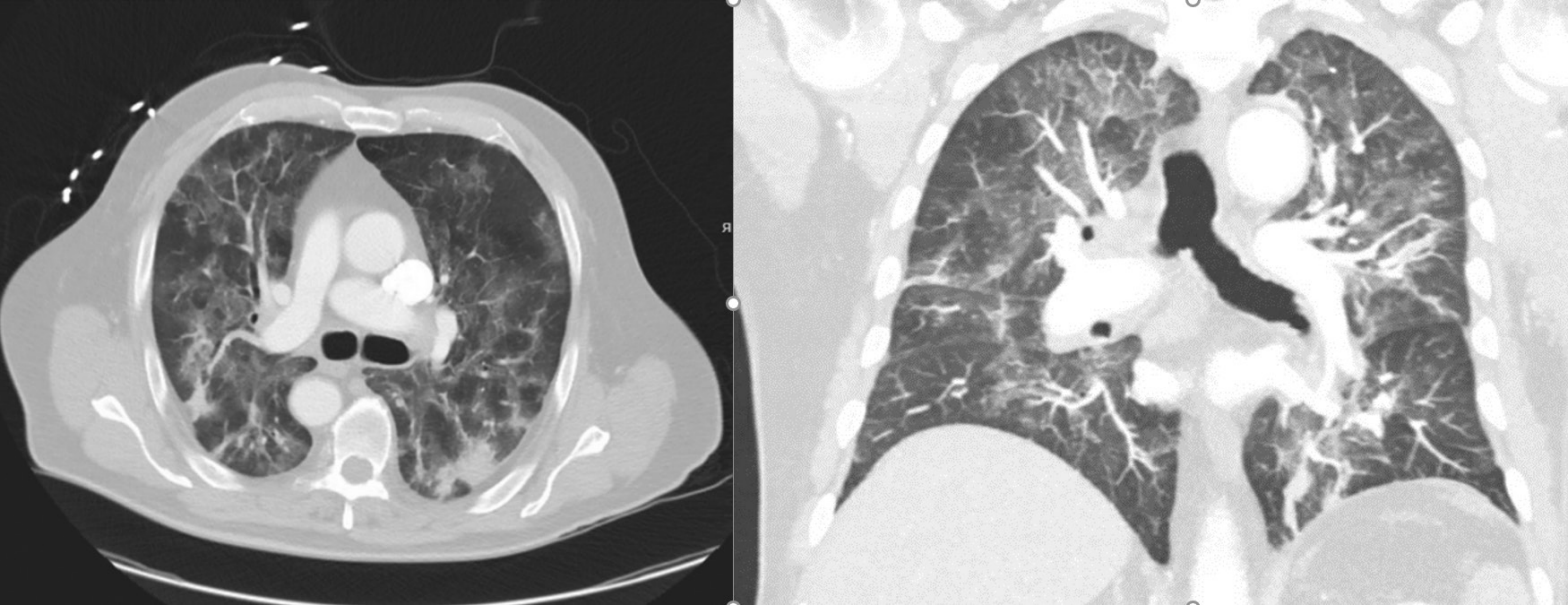
Representative slices of CT chest obtained on hospital Day 1 showed patchy ground‐glass opacity with areas of increased consolidation, especially in the left lower lobe.

A 75‐year‐old man presented to our emergency room and was admitted because of cough and worsening dyspnea on exertion for 4 days prior to presentation. His medical history was significant for mantle cell lymphoma diagnosed 10 months prior to admission, for which he received six cycles of Bendamustine, Rituximab, Cytarabine (BRCA) followed by maintenance Rituximab. His last chemotherapy was 3 months prior to, and last dose of Rituximab was 6 weeks prior to this admission, respectively. He had achieved a complete metabolic response based on recent Positive Electron Tomography (PET) imaging and blood work. On initial evaluation at emergency room, he was hypoxic requiring supplemental oxygen via nasal cannula, but otherwise his vital signs appeared well. Heart and lung auscultation normal. Computed tomography angiography (CTA) chest revealed widespread patchy ground‐glass opacities bilaterally compatible with multifocal pneumonia (Figure 1). Laboratory results revealed white blood cell count 3.2 K/mcL with 60% neutrophils, 20% bands, and 6% lymphocytes. Lactate dehydrogenase was elevated 1959 units/L (normal range 313–618 u/L), ferritin 1622 ng/mL (normal range 17.9–464.0 ng/mL), and D‐Dimer 1670 ng/mL (normal range <749 ng/mL). Nasopharyngeal swab for SARS‐COV2 polymerase chain reaction (PCR) was negative.

Urine antigen assay for *Streptococcus pneumoniae* and *Legionella pneumophila* was negative. Given his immunocompromised state, opportunistic pathogens including *Pneumocystis jirovecii* (PCP), and other fungal causes of infectious pneumonia, were considered, and serum beta‐d‐glucan testing and aspergillus antigen were sent. Method for Beta D Glucan (BDG) tests was Semi‐Quantitative Colorimetry using Fungitell kit produced by Associates of CAPE COD, INC. Aspergillus galactomannan Antigen test used Semi‐quantitative Enzyme Immunoassay. Both tests were carried out by ARUP laboratories. Drug‐related hypersensitivity pneumonitis or lung injury due to Rituximab were also considered but felt less likely given the timing of presentation. The nasopharyngeal swab was positive for adenovirus by multiplex respiratory virus PCR panel. He was started on empiric antibiotic therapy with ceftriaxone 1 g intravenous once daily and azithromycin 500 mg intravenous once daily for possible community‐acquired pneumonia and admitted to the medical ward for ongoing care.

On hospital Day 4, his hypoxemia worsened. He was transitioned to high flow nasal cannula, and initiated on dexamethasone 6 mg intravenous once daily based on dosing per RECOVERY trial^5^ for potential viral‐induced acute respiratory distress syndrome (ARDS), and he was escalated to the intensive care unit(ICU). At the time of upgrading to ICU level of care, the patient's Beta‐D‐Glucan test resulted positive with high level of >500 pg/mL. Provided CT chest findings and worsening hypoxemic respiratory failure, Trimethoprim‐Sulfamethoxazole (TMP‐SMZ) tab was initiated for high suspicion of PCP pneumonia with dosing of 1600–320 mg three times daily. Bronchoscopic sampling was not obtained for PCP staining and PCR confirmation due to tenuous respiratory status rendered the patient incapable to tolerate bronchoscopy. Serum Aspergillus galactomannan antigen was negative.

On hospital Day 5, patient developed hematuria, initially mild and thought to be due to traumatic Foley catheter insertion. However his hematuria gradually increased in quantity to the point of requiring continuous bladder irrigation. Urinalysis with culture and ultrasound of urinary tract were obtained without evidence of urinary obstruction/calculi, or bacterial urinary tract infection. Concern was raised for hemorrhagic cystitis which was due to disseminated adenoviral infection, and serum adenoviral PCR testing was sent to evaluate the viral load. On hospital Day 7, transaminases started trending up, with ALT 203 U/L and AST 176 U/L with negative findings on acute hepatitis panel. The patient also developed declining renal function with creatinine rising to 1.5 mg/dL (baseline creatinine 0.9 mg/dL) in the context of stable hemodynamics. The constellation of findings of interstitial pneumonia, transaminitis, and gross hematuria with acute kidney injury further supported a diagnosis of disseminated ADV infection. Dexamethasone was therefore held, and the patient was given intravenous immunoglobulin for ADV based on limited data.^6^, ^7^ He was not given Cidofovir at this time due to worsening renal injury. Serum BDG was repeated on Day 7 too.

**FIGURE 2:**
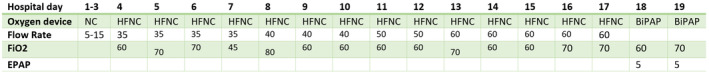
Daily progression of supplemental oxygen requirements. NC, nasal cannula; HFNC, high flow nasal cannula; BiPAP, bilevel positive airway pressure; EPAP, expiratory positive airway pressure; FiO2, fraction of inspired oxygen

**FIGURE 3:**
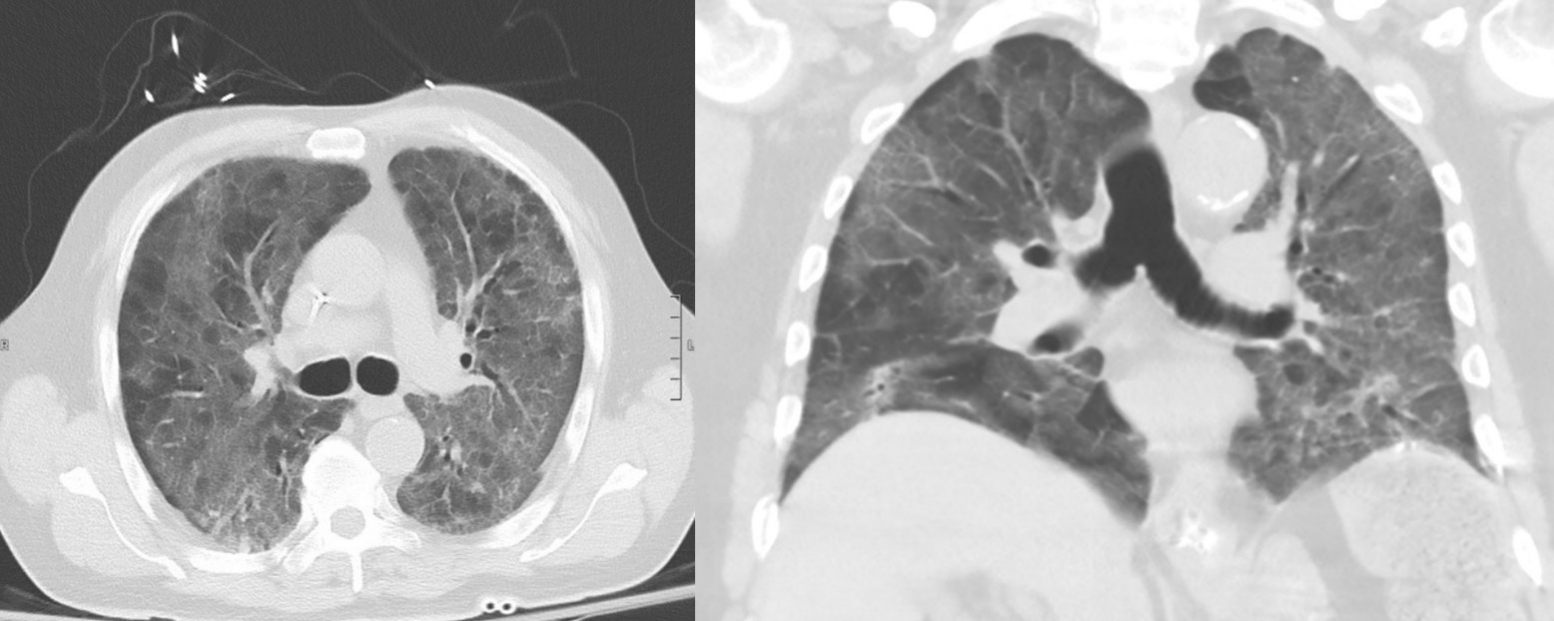
Representative slices of CT chest obtained on hospital Day 12 showed more diffuse ground‐glass opacities with peripheral clearing and reticulations consistent with organization and early fibrotic changes

The patient completed a 5‐day course of ceftriaxone and azithromycin for empirical coverage of suspected concomitant bacterial pneumonia. Ttrimethoprim/sulfamethoxazole were continued for PCP pneumonia given repeat BDG was positive again on Day 7 with a high level of >500 pg/mL, but switched to IV form with dosing of 600 mg twice daily due to lack of clinical response to tabs and patient's inability to take P.O on Day 10. Over the next week, patient's clinical status gradually declined despite broadening antibiotics to piperacillin‐tazobactam 3.375 g intravenous every 8 h and vancomycin, with the need for increasedsupplemental oxygen via high flow system (Figure 2), worsening renal failure necessitating renal replacement therapy, and increasing abdominal distension with adynamic ileus on CT abdomen. On hospital Day 12, whole blood adenovirus polymerase chain reaction (PCR) that had been sent 7 days earlier returned with elvated viral load of 548,000 copies/mL. Diagnosis of disseminated adenovirus infection was therefore confirmed. Repeat chest imaging at this time showed progression of multifocal ground‐glass opacities and evidence of organization and early fibrotic changes (Figure 3). The decision was made to start Cidofovir therapy after balancing risks and benefit in context of his acute kidney failure, with the dosing of  3 mg/kg, administrated twice weekly, in conjunction with probenecid 2000 mg po once as loading dose followed by 1000 mg po every 6 h. His hypoxemic respiratory failure continued to progress despite aforementioned medical therapy, high flow nasal cannula support, as well as continuous renal replacement therapy, requiring BiPAP that was started on hospital day 18. Patient was resumed on dexamethasone as salvage therapy for ARDS and for the unresolved PCP pneumonia.

Unfortunately, over the following days patient's mentation declined likely due to Central nervous system (CNS) involvement by ADV infection (CNS imaging was not obtained based on goals of care discussion). He was transitioned to full comfort care and passed away shortly on hospital Day 20. Repeat adenovirus PCR earlier in the same day morning revealed a viral load of greater than 10,000,000 copies/mL (>7 log copies).

**FIGURE 4:**
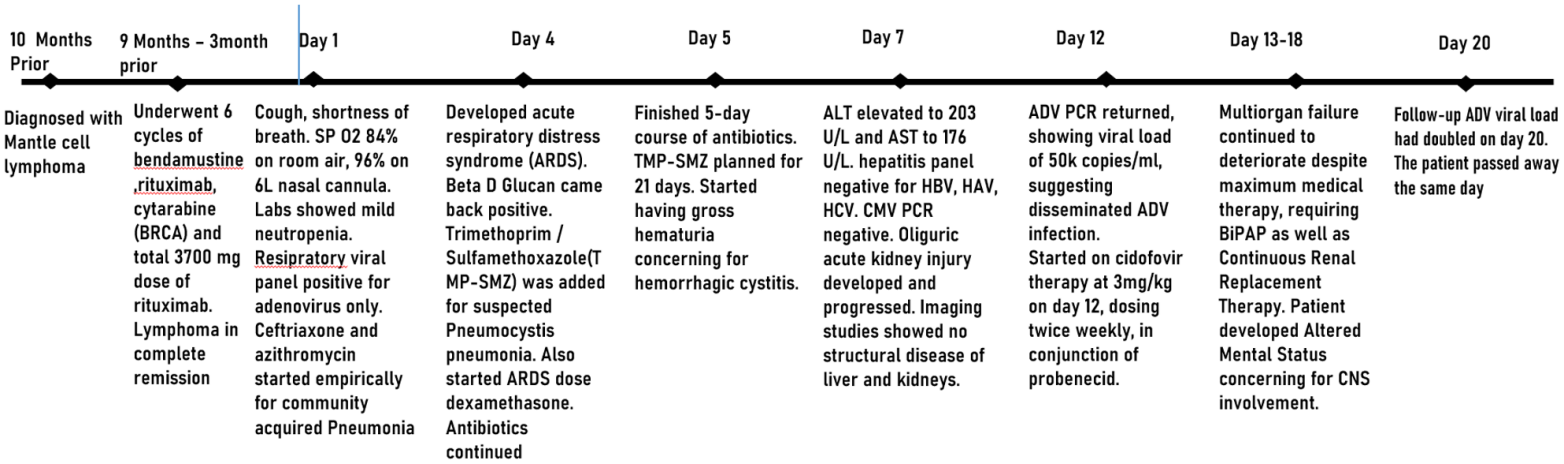
Time line of clinical findings, treatment, and course. ALT, alanine transaminase; AST, aspartate transaminase; CMV, cytomegalovirus; ADV, adenovirus

The time line of this case is illustrated in Figure 4.

## 3 DISCUSSION AND CONCLUSION

Adenovirus is a common pathogen associated with upper and lower respiratory tract infections, as well as viral conjunctivitis. The viral infection is usually self‐limiting, although devastating disseminated infections in immunocompetent individuals have been reported.^8^ Disseminated adenovirus infection usually occurs in immunocompromised hosts, such as those who underwent hematopoietic stem cell transplant (HSCT), solid organ transplant (SOT), or patients with acquired immunodeficiency syndrome (AIDS). Disseminated disease can manifest in the form of interstitial pneumonia, hepatitis, meningoencephalitis, and tubulointerstitial nephritis.

The incidence of adenovirus infection in patients who underwent HSCT can be highly variable (3–47%),^9^, ^10^ and disease is usually limited to one organ system (gastrointestinal, genitourinary, or respiratory system), and disseminated disease is considered if it involves two or more organs, with the exception for adenovirus viremia.^1^, ^11^ Based on hemagglutination traits, adenovirus species are divided into A‐G groups. Disseminated diseases in HSCT recipients appeared to be more frequently caused by adenovirus C species.^12^, ^13^ In SOT recipients, adenovirus infection is mostly allograft limited, though disseminated diseases are not uncommon.^14^

Conditioning chemotherapy before the HSCT is known to cause long‐lasting effects on the host's immune system due to depletion of host T/B cells, leading to increased risks of opportunistic infections. The reconstitution of immunity with engraftment is also a complex and high‐risk process related to the development of severe infections. T‐cell depletion by alemtuzumab or antithymocyte globulin is known to increase the risk of disseminated adenovirus.^15^

However, there are limited data about adenovirus infection in non‐HSCT/SOT patients who received only chemotherapy for hematological malignancies. Bendamustine has the features of both alkylating and antimetabolite chemotherapy agents. Thanks to its presumed safe side effect profiles, Bendamustine is often used off‐label to treat indolent lymphomas, such as follicular lymphoma and chronic lymphocytic leukemia. More recent data suggested Bendamustine can cause significant and prolonged CD4+ suppression of 24–26 months in a high percentage of patients who received it either as first‐line therapy or advanced treatment,^16^ leading to increased risk of both community‐acquired and opportunistic infections including adenovirus infection when compared to other chemotherapy agents for the treatment of indolent non‐Hodgkin Lymphoma in elderly patients.^17^

BDG is a useful diagnostic tool for PCP infection. In a meta‐analysis, the specificity of BDG for the diagnosis of PCP was reported to be 0.75 in HIV and non‐HIV patients combined (95% CI, 0.68–0.81), 0.78 and 0.73, for patients with and without HIV, respectively.^18^ PCP infection in the immunocompromised host has been well‐known, but co‐infection of PCP and adenovirus with severe systemic disease is extremely rare, with only one case report of such in an AIDS patient.^19^ Our patient may be the first reported case in those who only received chemotherapy for hematological malignancy. We must acknowledge we did not have directevidence to support the diagnosis of PCP such as PCP staining or PCR with bronchial alveolar lavage (BAL) due to patient's severe respiratory distress and consequent intolerance of bronchoscope, also patient was not producing any sputum with hypertonic saline induction. However, our case had two consecutive, strong positive BDG test results that make the diagnosis of PCP highly likely while the aspergillus was effectively ruled out by clinical, serological, and imaging findings. Of note, false positivity of beta‐D‐glucan can arise from exposure to cellulose dialysis membrane, albumin, immunoglobulins, and potentially some antimicrobials. One study had found very limited reactivity between the Beta‐d‐glucan assay and most commonly used intravenous antibiotics including ceftriaxone, TMP‐SMZ and Piperacillin‐tazobactam.^20^ In our case, the first beta‐D glucan was obtained before the administration of immunoglobulins (IVIG) and any intravenous antibiotics so false positive was effectively ruled out. Besides, the high level (>500 pg/mL) BDG further increased its validity. The second BDG test was collected 1 day after administration of IVIG and abovementioned antibiotics; however, again the high level is rather due to PCP infection than the cross‐reactivity to drugs.

Rituximab is associated with increased risks of hepatitis B, Varicella zoster virus, and Cytomegalovirus infections.^21^ In comparison, there is only one case of Rituximab‐related fulminant adenoviral hepatitis reported, who received 11,200 mg total dose of Rituximab for his lymphoma.^22^ Our patient's chemotherapy regimen consisted of Bendamustine, Cytarabine, and Rituximab (total dose of 3700 mg) and he did have lymphocytopenia at presentation though specific T/B cell subsets were not measured by flow cytometry that would be instrumental to provide more granular information of the adaptive immunity.

Of note, multiplex tools have limited sensitivity in general since they are designed to only detect common Adenovirus serotypes. Although being questioned about its value in the general patient population, it may still serve as a quick and convenient diagnostic modality in the diagnosis of possible opportunistic infections in immunocompromised patients such as in our case. In light of clinically severe disease, physicians may favor the management of more common infectious processes while diagnostic cues are weighted for multiple possibilities. In our case, this patient's positive BDG tests and relevant CT chest findings suggested PCP pneumonia, which was unfortunately drug resistant as evidenced by the strong positivity on repeat BDG test despite treatment with TMP/SMZ, likely due to severely impaired immune response and/or disseminated ADV.

Currently, there is no U.S. Food and Drug Administration (FDA)‐approved medicine for disseminated adenovirus disease. Nonetheless, Cidofovir is the standard treatment for disseminated adenovirus in many centers, but dosing regimens are center‐specific. There is no current study that compares the safety and efficacy of the two most frequently used regimens: 5 mg/kg every 12–2 weeks and 1 mg three times per week. Although a low‐dose regimen was associated with less nephrotoxicity, this may carry a higher risk of treatment failure and breakthrough infection.,^23^ Besides, Cidofovir has unpredictable effects as some patients seemed to respond well while others did not. BrinCidofovir is a lipid ester of Cidofovir with less nephrotoxicity than Cidofovir, which was reported to have a higher intracellular level crucial for virustatic effect. A few case reports showed the success of BrinCidofovir in the treatment of refractory adenovirus infection; thus, it may be a promising drug in the future, but more data are needed to confirm its efficacy. At this point, the availability of BrinCidofovir is also limited by the lack of approval by the FDA or its counterparts in many other locations in the world. Adoptive transfer of donor adenovirus‐specific T‐cells (ACT) is an experimental therapy with prompt response in HSCT, but this intervention failed to show the same benefits in solid organ transplant recipients.^24^ For patients deemed to receive chemotherapy as the only treatment for hematological malignancies, it is also unrealistic to routinely isolate and expand adenovirus‐specific T‐cells from each patient due to the low incidence of disseminated adenovirus infections in general and high cost of these procedures.

To conclude, disseminated adenovirus infections in patients with hematological malignancies who only received chemotherapy are rare, and co‐infection with drug resistant PCP is possible. Diagnosis of disseminated adenovirus infection (DAI) remains very challenging and treatment options are limited with unclear efficacy. Suspicion for DAI should be raised when interstitial pneumonia, hemorrhagic cystitis, acute liver injury, and other organ‐specific manifestations are present without an obvious cause, especially in the setting of a positive nasal PCR test for adenovirus. Disseminated adenovirus infection should be especially suspected in patients who received agents that can severely suppress T cell population, such as Bendamustine, Alemtuzumab, and Antithymocyte immune globulin. Cidofovir is currently the standard treatment for severe adenovirus infection, though the ideal regimen is unclear. Its derivative medication, BrinCidofovir, may offer better efficacy at less risk of nephrotoxicity; however, more data are required.

## AUTHOR CONTRIBUTIONS

**Bo Yu:** Investigation; writing – original draft. **Lakshmi Saravanan:** Writing – original draft. **Dena Hoa Tran:** Validation; writing – review and editing. **Alexander Davies:** Writing – review and editing. **Harpreet Kaur:** Supervision; writing – review and editing. **Shivakumar Narayanan:** Conceptualization; investigation; supervision. **Avelino Verceles:** Supervision; writing – review and editing. **Hyeong Kim:** Supervision.

## FUNDING INFORMATION

University of Maryland Health Sciences and Human Services Library's Open Access Publishing Fund.

## CONFLICT OF INTEREST STATEMENT

All authors declare that they have no competing interests.

## ETHICS APPROVAL AND CONSENT TO PARTICIPATE

Not Applicable.

## CONSENT

Written consent to publish this case report and any accompanying images was obtained from the patient's next of kin.

## Data Availability

Any data (suitably anonymized to maintain patient confidentiality) are available for readers to review if a suitable written request to the Corresponding author is made.
